# Proteomics and Metabolomics Approaches towards a Functional Insight onto AUTISM Spectrum Disorders: Phenotype Stratification and Biomarker Discovery

**DOI:** 10.3390/ijms21176274

**Published:** 2020-08-30

**Authors:** Maria Vittoria Ristori, Stefano Levi Mortera, Valeria Marzano, Silvia Guerrera, Pamela Vernocchi, Gianluca Ianiro, Simone Gardini, Giuliano Torre, Giovanni Valeri, Stefano Vicari, Antonio Gasbarrini, Lorenza Putignani

**Affiliations:** 1 Department of Laboratories, Unit of Parasitology and Area of Genetics and Rare Diseases, Unit of Human Microbiome, Bambino Gesù Children’s Hospital, IRCCS, 00165 Rome, Italy; mvittoria.ristori@opbg.net; 2 Area of Genetics and Rare Diseases, Unit of Human Microbiome, Bambino Gesù Children’s Hospital, IRCCS, 00165 Rome, Italy; stefano.levimortera@opbg.net (S.L.M.); valeria.marzano@opbg.net (V.M.); pamela.vernocchi@opbg.net (P.V.); 3 Department of Neuroscience, Unit of Head Child & Adolescent Psychiatry, Bambino Gesù Children’s Hospital, IRCCS, 00165 Rome, Italy; silvia.guerrera@opbg.net (S.G.); giovanni.valeri@opbg.net (G.V.); stefano.vicari@opbg.net (S.V.); 4 Dipartimento di Gastroenterologia, Università Cattolica del Sacro Cuore, Fondazione Policlinico Universitario A. Gemelli IRCCS, Largo A. Gemelli 8, 00168 Rome, Italy; gianluca.ianiro@hotmail.it; 5 GENOMEUP S.R.L, Viale Pasteur 8, 00144 Rome, Italy; simone@genomeup.com; 6 Department of Specialist Pediatricians and Liver-Kidney Transplantation, Bambino Gesù Children’s Hospital, IRCCS, 00165 Rome, Italy; giuliano.torre@opbg.net; 7 Department of Life Sciences and Public Health, Catholic University, 00153 Rome, Italy; 8 Istituto di Patologia Speciale Medica, Università Cattolica del Sacro Cuore, 00168 Rome, Italy; antonio.gasbarrini@unicatt.it; 9 UOC Medicina Interna e Gastroenterologia, Area Gastroenterologia ed Oncologia Medica, Dipartimento di Scienze Gastroenterologiche, Endocrino-Metaboliche e Nefro-Urologiche, Fondazione Policlinico Universitario A. Gemelli IRCCS, 00168 Rome, Italy

**Keywords:** autism spectrum disorders (ASDs), proteomics, metabolomics, interactomics, disease biomarkers, clinical decision support systems (CDSSs)

## Abstract

Autism spectrum disorders (ASDs) are neurodevelopmental disorders characterized by behavioral alterations and currently affect about 1% of children. Significant genetic factors and mechanisms underline the causation of ASD. Indeed, many affected individuals are diagnosed with chromosomal abnormalities, submicroscopic deletions or duplications, single-gene disorders or variants. However, a range of metabolic abnormalities has been highlighted in many patients, by identifying biofluid metabolome and proteome profiles potentially usable as ASD biomarkers. Indeed, next-generation sequencing and other omics platforms, including proteomics and metabolomics, have uncovered early age disease biomarkers which may lead to novel diagnostic tools and treatment targets that may vary from patient to patient depending on the specific genomic and other omics findings. The progressive identification of new proteins and metabolites acting as biomarker candidates, combined with patient genetic and clinical data and environmental factors, including microbiota, would bring us towards advanced clinical decision support systems (CDSSs) assisted by machine learning models for advanced ASD-personalized medicine. Herein, we will discuss novel computational solutions to evaluate new proteome and metabolome ASD biomarker candidates, in terms of their recurrence in the reviewed literature and laboratory medicine feasibility. Moreover, the way to exploit CDSS, performed by artificial intelligence, is presented as an effective tool to integrate omics data to electronic health/medical records (EHR/EMR), hopefully acting as added value in the near future for the clinical management of ASD.

## 1. Introduction

**Figure 1:**
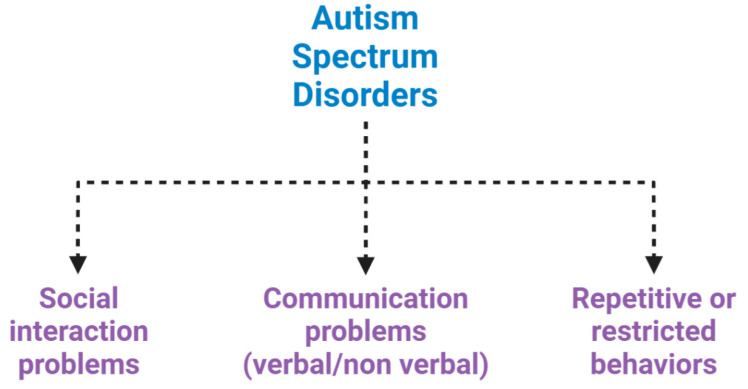
Neuropsychiatric features of autism spectrum disorder (ASD). ASD is characterized by impairments in social interaction, difficulty in adapting behaviour in various social contexts, or lack of interest in peers; communication problems, such as difficulty making eye contact, facial expressions, body postures, and difficulty understanding or using the gestures that regulate interaction with others; and restricted or repetitive behaviours, such as rituals that are conducted with a rigid manner or movements.

Autism spectrum disorders (ASDs) are a complex set of neurodevelopmental diseases, behaviourally diagnosed, that affect several spheres of mental development. They represent a panel of conditions that begin during the developmental period and result in impairments of personal, social, academic or occupational functioning (Figure 1) [1,2,3].

Comorbidity with intellectual disabilities, impaired motor coordination and gastrointestinal (GI) disorders are also often present [3,4]. An incidence of ASD of 1 in every 60 subjects is estimated in the United States, with a fourfold frequency for males with respect to females [5,6,7]. Clinical manifestations typically occur in the 2nd–3rd year of life, usually persist in adulthood, and influence various aspects of mental development [8]. The exact etiopathogenesis of ASDs is not yet well known but many studies have investigated both genetic and environmental factors [9,10]. In particular, gene inheritance has been found in around 60% [11,12] or 80% [13] of cases with significant heterogeneity concerning the genetic factors actually involved in ASD onset [14,15]. In a review of the literature, Higdon R. et al. found that many ASD patients have recurrent de novo disruptive mutations particularly affecting specific protein targets such as chromodomain helicase DNA binding protein 8 (CHD8), activity-dependent neuroprotector homeobox (ADNP), dual-specificity tyrosine phosphorylation-regulated kinase 1A (DYRK1A), and phosphatase and tensin homolog (PTEN). In particular, subjects with PTEN mutations showed abnormal brain white matter volumes in addition to autism symptoms; subjects with CHD8 mutations carried chronic GI complications, distinct facial dysmorphology and macrocephaly; those with DYRK1A mutations had a higher probability of microencephaly and early growth difficulties compared to healthy subjects; and patients carrying an ADNP-disruptive mutation were characterized by intellectual disability and dysmorphic features [16]. Interestingly, a recent study directed by Risch and his group found that ASD siblings showed a risk increment of 10.1% to develop ASD, compared to control siblings, hence confirming a genetic predisposition in the familial recurrence of ASD [10]. Additionally, oxidative stress, inflammation, mitochondrial dysfunction, and immune dysregulation, associated with changes in protein and metabolites pathways, have been reported in the literature in many ASD studies [17,18,19].

**Figure 2:**
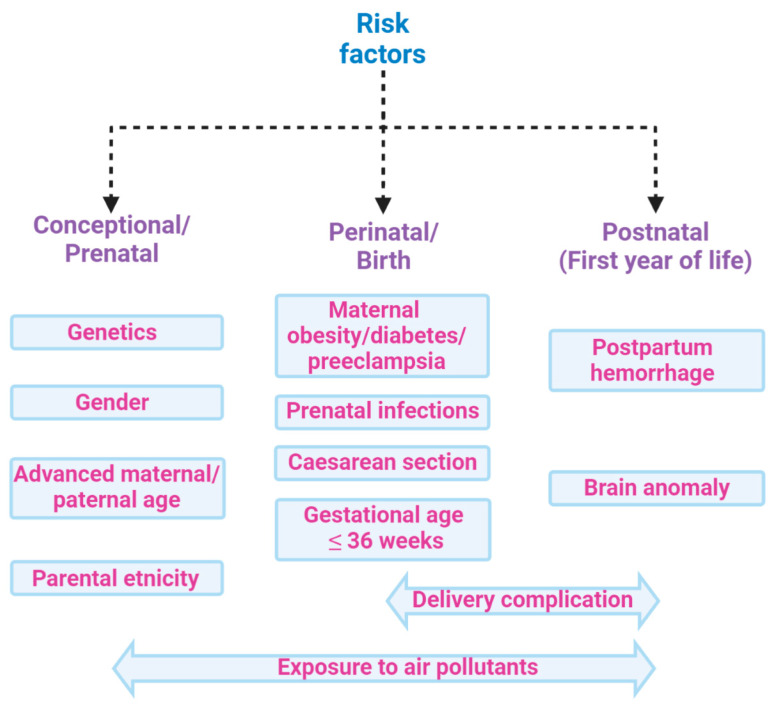
Risk factors associated with ASD: ASD is a multifactorial condition characterized by genetic and environmental factors, including prenatal and postnatal factors that increase the risk of disease. Among the main factors, genetic predisposition, parents’ age, and exposures during pregnancy to air pollutants have been associated with poor cognitive outcomes in the perinatal age. Moreover, delivery complications or postpartum haemorrhage might also increase the risk of ASD. All these factors, globally constituting the exposome, may contribute to ASD, hence hampering the search for single biomarkers of the disease.

However, it is also well established that several factors involving the pre-, perinatal, or postnatal environment are associated with amplified risk of ASD [20] (Figure 2). Amongst these, the main factors of maternal infections, stress, and diabetes, also in addition to exposure to pesticides, air pollution, dietary habits, infections, inflammatory conditions, and consumption of antibiotics during pregnancy, which together make up the so-called exposome, combine to increase the ASD risk [21,22]. Indeed, environmental factors can influence genetic vulnerability [23] by modifying the development of neuronal circuits [24]. These alterations are heterogeneous and this can contribute to determining the complexity of the disorder in terms of neurobiology, symptomatology, and etiology [25]. In particular, GI comorbidity associated with an alteration in the microbiota composition is frequently reported in ASD groups. [21,26]. Indeed, many studies have investigated the association between autism severity and GI, showing positive correlations [26,27,28,29,30]. The increased presence of irritability [31], anxiety and affective disorders [3,32], dysregulation and externalizing problems [33], rigid/compulsive behaviors [34,35], increased sensory sensitivity [36], and sleep problems [33,37] have been reported in ASD subjects with concurrent GI symptoms compared to ASD subjects without. Significant GI symptoms are associated with chronic abdominal pain [3,4,26,33,38,39], chronic constipation [3,4,26,39,40], chronic diarrhea [3,39,41], and gastroesophageal reflux [26,38,42]. The existence of a complex bidirectional interaction between the central nervous system and the GI tract is currently more than a hypothesis and primarily involves the impact of ecology and function of the microbiota [43].

Targeted-metagenomics studies on the ecology of the microbiota, performed using next-generation sequencing (NGS), have revealed that specific signatures, such as *Prevotella*, *Enterococcus*, *Lactobacillus*, *Ruminococcus*, *Faecalibacterium prausnitzii*, *Sutterella*, and *Bifidobacterium*, are overrepresented in children with ASD compared to healthy controls [28,44,45,46]. Furthermore, metabolomics studies evidenced an involvement of the microbiota in the production of molecules, such as tryptophan, inflammatory cytokines, or cortisol, which exert a role in ASD disorders and GI-related symptoms, as recently reported by our group [47]. Therefore, an approach to the investigation of ASD based on omics or meta-omics disciplines is becoming crucial for both disease phenotype stratification and biomarker discovery. Given the large amount of data (i.e., big data) produced by a single omics or meta-omics discipline, an untargeted evaluation of data may provide substantial new models to properly stratify a multifactorial disease such as ASD, considering both host and microbiome profiling and producing integrated models to co-represent genomics/metagenomics, metabolomics/metametabolomics, and proteomics/metaproteomics profiling of the disease. 

In particular, transcriptomics and proteomics may improve the existing gene models by profiling molecular phenotypes at transcriptionally active regions of the genome (the transcriptome) and protein abundance (the proteome) levels. Besides a proteomics profiling, post-translational modifications and protein–protein interactions can contribute to providing further specific information on pathways and molecular networks. Furthermore, metabolomics may provide information on the modulation of the host and microbiota metabolism. As previously reported, the diagnosis of ASD depends on clinical observation and procedures to evaluate behavioral, historical, and parent-report information [48]. These tools may involve a significant degree of host variability; hence, detecting metabolomics biomarkers may contribute to the advance of the diagnostics and clinical management of ASD, and may provide new biomarkers that could be used to improve the outcome of individualized interventions as personalized medicine tools.

Given the growing interest in identifying new functional traits of the disease and novel biomarkers, big data obtained by metabolomics and proteomics approaches from blood, urine or saliva specimens should be collected and stored in open source digital biobanks available to omics scientists and clinicians for deep phenotyping. This system biology-based approach will allow multidimensional data to be integrated at cellular, tissue and organ organization levels, providing computational chemometric models concurring in the understanding of the pathophysiological mechanisms of diseases (i.e., onset and progression) [49]. In this paper, we introduce a new analysis of the data (i.e., ontology enrichment of protein and metabolites), based on the category “Biological Process” to highlight new possible biomarkers and metabolic pathways that could open new avenues to the study of ASD disease biomarkers based on proteomics and metabolomics. Moreover, novel biomarkers are discussed in terms of their added value in clinical decision support systems (CDSS), approached by artificial intelligence (AI), that represent a new way to integrate omics, multi-omics data and health/medical records (EHR/EMR). This may represent a novel tool to assist clinicians in the near future to approach ASD phenotype stratification and hopefully to disentangle the complexity of this disease due to multifactorial components.

## 2. Methods

### 2.1. Search Strategy

We conducted a review of the literature to evaluate the role of the proteomics and metabolomics disciplines, and data for disease phenotype stratification and biomarker discovery, in ASD patients. The research was conducted on PubMed, including papers from 2004 to 2019 and using the following terms: “autism” or “autism spectrum disorder” and “proteomics” or “interactome” or “metabolomics” or “protein” or “metabolites” and “omics”. All articles providing enough information about the relationship between multi-omics data and ASD were included.

### 2.2. Selection Criteria

The inclusion criteria for study selection were the following: (1) observational prospective and retrospective studies, case–control studies, cohort studies, or systematic reviews; (2) studies involving proteomics- and metabolomics-based biomarker research in ASD; and (3) English written studies. All studies that did not meet the following criteria were excluded from the review process.

### 2.3. Analysis of Protein and Metabolites Highlighted in Table 1 and Table 2

New “ontology enrichment” analyses, based on class, subclass, and biological processes were performed for both metabolites and proteins reported in Table 1 and Table 2. The aim was to identify new potential biomarkers found in the literature that could be interpreted in a new way for appropriate evaluation in decision support systems (DSSs) through the framework discussed herein.

To be precise, we analyzed 140 proteins, as shown in Table 1:

1. we downloaded all the information from the UniProt database [80], via a Perl script;
2. we analyzed all of the Gene Ontology terms for “Biological Process” (GOTERM-BP) [81];
3. we calculated the terms found through ad hoc scripts in bash;
4. we designed a bubble chart by taking GOTERM-BP with the source “Traceable author statement” (e.g., TAS: Reactome) starting from the highest value (count) and then descending ending the list of proteins. Thus, we clustered all of the proteins for GOTERM-BP with at least one term.

Some proteins were not included in the analysis because we did not find the associated GOTERM-BP TAS.

We have analyzed 119 metabolites, as shown in Table 2:

1. we started from the complete Human Metabolome Database (HMDB) [82];
2. we selected our metabolites, analyzed the classes, subclasses and all terms under the Ontology category and Biochemical Pathway terms, such as biological process and physiological effect, via an ad hoc script in Python;
3. we calculated the terms found through ad hoc scripts in Python;
4. we designed a bubble chart by taking all of the associated biological processes.

Some metabolites were not included in the analysis because we did not find the associated ontology terms in HMDB.

## 3. Stratification of Complex Disease Phenotypes, Early Interventions and Omics-Generated Biomarkers

There is high variability in ASD symptoms and severity, principally focused on a general deficit in interpersonal interactions, due to reduced or absent verbal communication, or difficulty in communicating with other people [83]. This heterogeneity could result from a combination of many molecular mechanisms and environmental issues and an integrative approach could be a new strategy for ASD deep profiling, possibly allowing behavioural intervention as quickly as possible. Indeed, some studies showed a significant improvement when therapies started before 24 months of age, at which time the neural system is still extremely mouldable [84]. Early interventions would probably reduce the cognitive abnormalities of subjects and help in preventing the onset of an autistic condition [85]. Thus, early diagnosis and prediction are essential for ASD and could be achieved due to the study of molecules involved in the etiopathogenesis of the ASD phenotype. Subgroups can be individuated in ASD patient cohorts, including functioning levels based on different parameters such as IQ values, language, and/or reading impairment, and this could help in the identification of gene/protein candidates or molecular mechanisms that can be associated with one or more of these subpopulations. This type of approach could be useful for the diagnosis and prognosis of the disease and/or for the choice of the therapy to be applied [86,87,88,89,90].

Omics and meta-omics disciplines currently allow deep investigation of ASD starting from a wide panel of samples, ranging from biological fluids to tissues. Omics sciences originate from a holistic vision of the system under study, thus overcoming the classical genetic/biochemical studies based on single or few target molecules. The strength of omics approaches resides in their ability to provide complete profiles of biological “features” (genes/transcripts/proteins/metabolites) to obtain a broad description of a biological system by the so-called systems biology. A non-targeted and high-throughput search of the genetic scaffold (DNA) and functional reservoir (RNA, proteins, metabolites) is key to decoding the pathophysiology of systems as complex as biological systems without any “a priori” statement on the descriptive variable of the systems, hence providing the most appropriate stratification of the disease or identification of novel biomarkers.

These new approaches are possible due to high-throughput technological platforms that have been developed with unprecedented specificity and sensitivity, fused to modern data processing based on the most recent bioinformatics and computational tools, and capable of processing the big data produced. Indeed, the current challenges of the omics technologies rely on the harmonization and integration of the big data generated by the different technologies [91].

**Figure 3:**
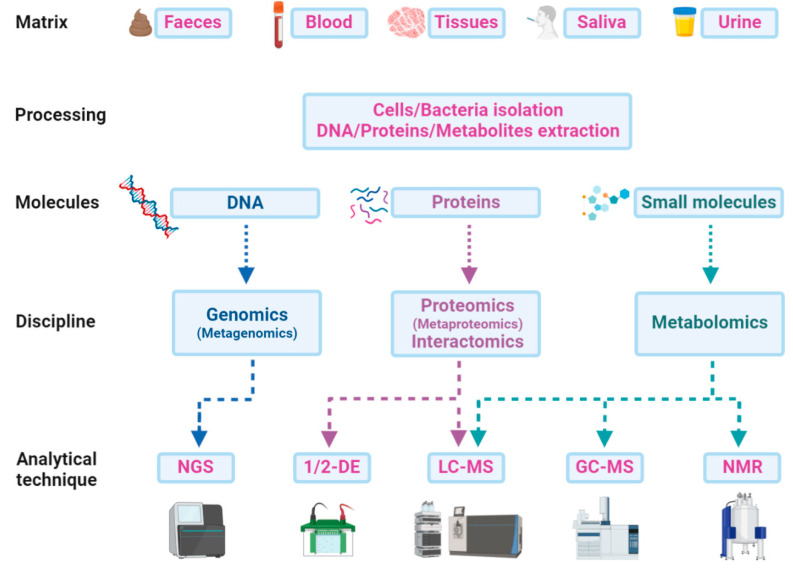
Scheme of the paths from matrices to analytical techniques in a multi-omics approach: An integrative approach could be a new strategy for ASD deep profiling, which combines data from genome sequencing (next-generation sequencing—NGS) with those from proteomics and metabolomics by one or two-dimensional gel electrophoresis (1/2-DE), liquid-chromatography and gas-chromatography mass spectrometry (LC-MS or GC-MS), or even from metabolomics data obtained by nuclear magnetic resonance (NMR) experiments.

A multi-omics approach that combines and integrates the results of more than a single discipline is becoming crucial to understand the pathophysiology of ASD and to identify new diagnostic and prognostic biomarkers from blood, saliva, urine, faeces, or other body fluids [19]. Different matrices can be treated for the direct extraction of DNA, proteins, peptides, or metabolites, or preliminarily processed for fractionation and isolation of cells or bacteria (Figure 3). Genomics or metagenomics follow a single path, starting with the DNA extraction from any matrix and finishing with the genome sequencing and the following bioinformatics’ pipeline. Proteins can also be extracted from a wide range of samples and analysed by liquid chromatography and mass spectrometry (LC-MS). Gel electrophoresis alone does not provide the same performance in biomarker discovery and is often used as a further preliminary fractionation step before LC-MS. In this sense, interactomics is a powerful tool for focusing on a potential target and its relationships with other proteins in promoting or inhibiting important functions related to ASD. Faeces and saliva are the most frequently used matrices for bacterial protein extraction, which is a crucial step in microbiota metaproteomics. The subsequent analytical procedure does not differ from that of a study on a single proteome but everything concerning data analysis or taxonomic assessments must rely on huge computational efforts for the individuation of a microbial biomarker or a functional signature of the microbiota or of the host co-metabolism. Metabolites can be easily extracted from faeces along with all biological fluids and analysed with different techniques on the basis of the chemical–physical features of molecules. The volatile fraction of metabolites can be detected by gas chromatography-mass spectrometry (GC-MS) experiments for both untargeted profiling and targeted quantitative analysis. Other small molecules can be analysed by NMR, LC-MS, or even direct infusion in MS.

A crucial point regards “sample size” computation (i.e., statistical power) for big data that cannot be predicted based on “basic” statistics but rather uses multivariate and univariate analyses for multi-step reduction of multidimensional data. Indeed, big data are usually produced by considering an order of tens or hundreds of samples, but this point (i.e., the number of samples) can be established for each produced chemometric model using different multivariate statistical methods (e.g., Hotelling T2, number of misclassifications (NMC), area under the receiver operating characteristics curves (AUROC), and discriminant Q2; in the case of the latter, in contrast to NMC and AUROC, PLS-DA models with low complexity compared to PLS models are preferred). Therefore, each integrated model based on omics-derived data needs to be validated for each sample set under analysis [92,93,94,95].

Integrated multi-omics approaches usually involve metabolomics in conjunction with genomics or metagenomics. However, the coupling of metabolomics and proteomics, in which both yield information about the functional aspects of ASD, appears to be even more promising. Thus, this review mainly focuses on these disciplines. 

### Computational Models Used to Generate Proteomics and Metabolomics Single and Fused Data

Regarding computational pipelines (“dry laboratories”) able to process big data generated from omics platforms (“wet laboratories”), the analysis of single and fused multidimensional data requires several steps of multi-step reiterated reduction supported by multivariate and univariate models. The variables under study may be the number of subjects (i.e., healthy or patients) and their omics “features” (i.e., proteins, metabolites), represented by worksheets that, overall, represent the system under study; such a system can be represented by a different number of features expressible by different percentages (e.g., 80–50–20%). Hence, the representativeness of the system (e.g., subjects and omics variables) can be evaluated by considering the most appropriate percentages to depict the entire system. Therefore, the starting raw data matrix (big data) becomes reduced to a new data matrix (smart data), the latter ready to be harmonized and integrated into a fused omics data model with data coming from different omics platforms. Then, the reduced and integrated data can be analyzed by chemometric models based on different multivariate statistical methods, as previously described [96]. In this way, the integrated generated model can be validated for each type of sample set (e.g., row data from GC-MS, LC-MS, ^1^H-NMR, etc.) and for each patient and/or reference subject dataset (e.g., phenomics data) [92,93,94,95].

## 4. Translational and Clinical Proteomics

The rapid expansion of proteomics in recent decades has provided powerful tools to undertake investigations of biological systems, aimed at identifying biomarkers for clinical diagnosis, monitoring the stage of diseases, studying the pathogenetic molecular mechanism, and choosing appropriate treatments.

Translational proteomics is a crucial component of a picture in which other omics and meta-omics disciplines (genomics, metabolomics/lipidomics, transcriptomics, and microbiomics), contribute to complex workflows producing both qualitative and quantitative relevant outcomes.

Under the approach of omics research, molecular profiles, combined with clinical profiles of patients, can be managed to drive clinical decisions, and, hence, advanced treatments. However, a key challenge is to overcome technical bottlenecks and to bridge the gap between early-stage discovery (translational research) and the subsequent stage, which is represented by routine quantitative searching and the determination of biomarker candidates in clinical research settings. Moreover, to accelerate the discovery of clinically actionable biomarkers, the focus must be switched from identification (ID) to quantitation. That is, precision proteomics must converge with precision medicine, in addition to other omics sciences. 

For this purpose, the major issue of quantitation and calibration in mass spectrometry (MS) must be correctly addressed. During the past decade, numerous experimental strategies have been developed and refined, together with tools and analytical kits, to provide researchers with a number of solutions for different proteomic approaches, on the basis of sample complexity and matrix variability. These approaches are difficult because a series of factors must be considered for both relative and absolute proteomics quantification [97]. Given the ascertained performance of MS in the absolute quantification of proteins, as a strong alternative to immunoassays, the principal needs currently appear to be focused on the calibration methods, not only to improve the measurement precision but also to make data interchangeable by laboratories globally. Synthetic stable isotope labelled (SIL) proteins and peptides, used as internal standards (IS), present both advantages and drawbacks, due to availability, cost, reproducibility of biological features, and accuracy [98]. An open question is how recombinant SIL proteins, synthetic labelled tryptic peptides (tSIL), or even the so-called “flanked” or “winged” peptides, could be used with reasonable confidence to provide the best accuracy as an internal calibration (IC) in bottom-up experiments [99]. Furthermore, new strategies for external calibration (EC), by means of external reference (molecules), are needed for selected or multiple reaction monitoring experiments (SRM/MRM), to correct bias coming from instrumental variance and sample preparation workflows [100]. Concerning protein ID, due to the continuous growth of public repositories of MS datasets, the use of spectral libraries is increasingly mined compared to classical peptide sequences matching with protein databases (DB). Data-independent acquisition (DIA) approaches appear particularly promising for future applications in biomarker discovery in proteomics. SWATH (Sequential Window Acquisition of all Theoretical Mass Spectra) analysis is currently employed for large scale ID and quantitation in proteomics, with the aim of exploiting the increasing number of spectral libraries available, considerably reducing computational time and space [101]. Applications in metaproteomics remain limited but the desire to identify alternative paths for protein detection and inference, overcoming the bias of conventional sequence databases, makes this an intriguing analytical strategy for faster identification/quantitation [102,103].

The extension to metabolomics continues to suffer from a lack of public libraries, but also appears to be a powerful approach. A new tool has recently been developed to match MS and MS/MS spectra searching for identical or analogous features in public repositories of metabolomics data [104].

Fewer proteomics studies on ASD have been published than those based on other disciplines, such as genomics or transcriptomics. Many of them rely on post-mortem brain tissues [50,51,52], serum [53,54,55,56], plasma [57,58,59], urine [63], saliva [60,61,62] direct samples or lymphoblastoid cell lines [64] (Table 1).

The first study on brain tissues was conducted by Junaid et al. [50]. Post-mortem brain samples from 8 ASD patients and 10 controls were analysed using two-dimensional gel electrophoresis (2-DE) followed by liquid chromatography-tandem mass spectrometry (LC-MS/MS) analysis; the glyoxalase 1 (Glo1) protein showed a shift in net charge allowing a polymorphic form of the protein in the brain of ASD patients to be characterized. Furthermore, they found that isoform have reduced enzyme activity and its variant was found to be increased in ASD patients with respect to control cases. The authors suggested that the homozygosity of a Glo1 variant could be one of the susceptibility factors in the etiology of autism. In another study of post-mortem brain tissue, the authors studied the prefrontal cortex of 10 ASD individuals and the cerebellum of 16 ASD patients versus control subjects. The results showed that the proteins, found in the brain tissue of ASD patients, are involved in processes of synaptic vesicle regulation, myelination, and energy metabolism [51]. Recently, Wingo et al. recruited 104 participants who were followed for up to 14 years, and 27% of the cohort were finally diagnosed as ASD. They studied proteins to identify new processes underlying variation in cognitive modification, by label-free LC-MS/MS experiments and found 579 proteins associated with cognitive trajectory after meta-analysis. Moreover, they found 38 proteins related to cognitive trajectory independently of β-amyloid plaques and neurofibrillary tangles [52]. Studies conducted on non-neurological tissues highlighted changes in quantities of proteins associated with inflammation or regulation of the immune system, including some interleukins [50,51,105,106,107,108]. Interestingly, most proteomic studies investigating ASD identified proteins involved in lipid metabolism and differentially expressed in ASD [51,55,57,105,106,109,110]. As an example, Corbett et al. identified differentially expressed peptides using LC-MS/MS, which allowed for the recognition of complement factor H-related protein (FHR1), apolipoprotein (APO) B-100, fibronectin 1 (FN1), and complement C1q as dysregulated proteins in children with ASD compared to control subjects [53]. In another study, apolipoproteins involved in cholesterol metabolism were found to be increased in an ASD cohort with respect to the control subjects [54]. In addition, two studies reported an alteration in proteins implicated in lipid metabolism, in the inflammation process and cell growth [55,56] (Table 1).

The study of Cortelazzo evidenced a total of 12 dysregulated proteins, associated primarily with acute inflammatory response [57]; despite irregularities of lipid metabolism shown in ASD tissues, the authors could not characterize primary or secondary occurrence in ASD pathophysiology. In the study by Pichitpunpong et al., ASD patients were subdivided into subgroups using the Autism Diagnostic Interview-Revised (ADI-R) method, and subsequently, dysregulated genes were identified by studying transcriptome profiles of individuals with ASD and control subjects. They profiled protein from lymphoblastoid cell lines using 2-DE followed by LC-MS/MS. Subsequently, proteins were compared to the dysregulated transcripts. Selected proteins were also analysed by Western blotting [64]. The authors reported 82 proteins linked to subjects with ASD and with severe language impairment and, among these, 14 were correlated with inflammation and neurological functions. Moreover, the diazepam-binding inhibitor (DBI) protein was notably reduced in the subgroup with severe language impairment. Furthermore, its expression levels were matched with the ADI-R items [64]. Recently, Shen et al. used the iTRAQ-based proteomics approach to compare plasma protein profiles of ASD compared with healthy subjects. They identified 24 differentially expressed proteins expressed in different pathways associated with ASDs [59] (Table 1). This evidence supports the thesis that synapsis growth, the complement system, cytoskeleton-related activities and cell adhesion are all involved in ASD. Moreover, using ELISA (enzyme-linked immune-adsorbent assay) and ROC (receiver operating characteristic) analysis, the authors found five proteins as possible biomarkers to discriminate ASD from controls [59]. Interestingly, they identified focal adhesion, cell adhesion molecules, and leukocyte trans-endothelial migration pathways that were correlated with ADS in a Chinese cohort [111].

Saliva, urine, and blood samples have also been taken into consideration. In particular, saliva-based studies showed an alteration in the processes involved in antimicrobial peptides, immune response, and inflammation at the mucosal level [60,61,62].

Overall, a large number of studies highlighted abnormalities in synapse biology in ASD patients, showing a prevalence of proteins related to neural tissue, and evidencing a limitation in the application of non-neural tissues in proteomics studies of ASD.

Indeed, due to the blood–brain barrier (BBB), it can be difficult to find proteins associated with ASD in peripheral blood, as possible disease-specific protein markers, despite the ease of use, cost-effectiveness, and low invasiveness of blood collection. Moreover, a study highlighted high intestinal and BBB permeability in ASD patients; indeed, ASD shows a reduced expression of occluding, tricellulin, claudin-1, and increased pore-forming claudins, which are part of gut barrier-forming “tight junction” (TJ) components [108]. Furthermore, it was found that a protein (zonulin) involved in intestinal permeability increased in ASD patients compared with controls, particularly with respect to the Childhood Autism Rating Scale score. [109]. Thus, possible blood biomarkers could be proteins associated with intestinal and BBB permeability. 

**Figure 4:**
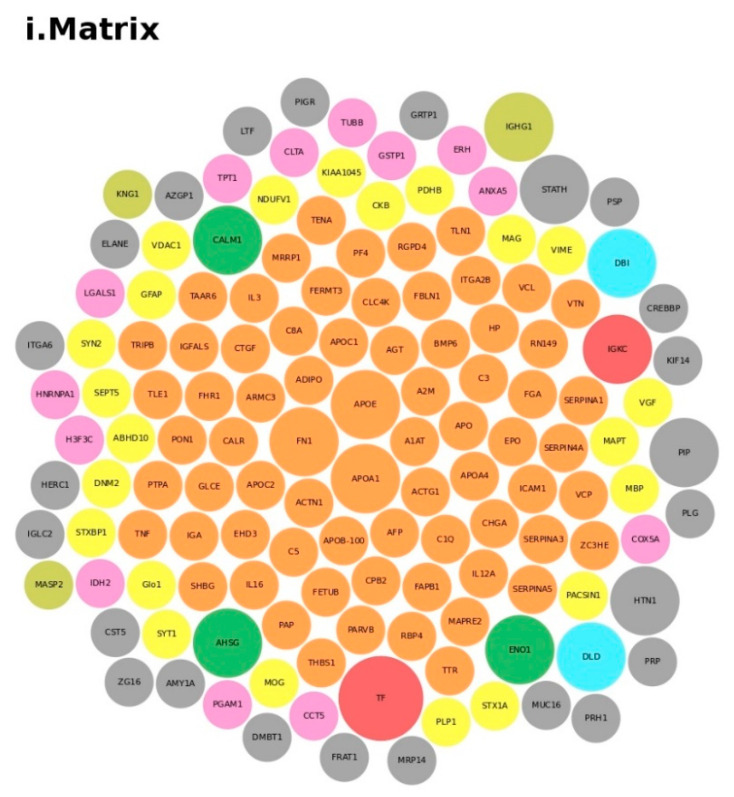

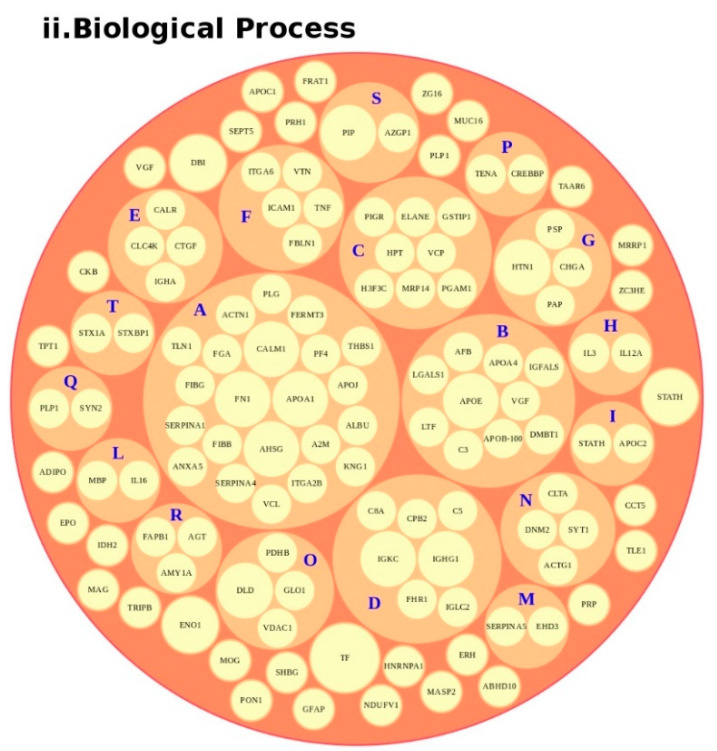
Protein analysis as a tool for the decision support system (DSS). In box (**i**), we grouped the proteins highlighted in Table 1 and Table S1 according to the matrix in which the proteins were studied, such as blood (**orange**), blood and urine (**lawn green**), brain (**red**), dried blood (**brown**), urine (**pink**), and brain biopsies, urine and blood (**lilac**). The size of the bubbles indicates the number of times the protein was found in that matrix in the different studies taken into consideration. In box (**ii**), we analyzed the data for the biological process and clustered the protein. Legend code: (A) platelet degranulation (GO:0002576); (B) cellular protein metabolic process (GO:0044267); (C) neutrophil degranulation (GO:0043312); (D) regulation of complement activation (GO:0030449); (E) receptor-mediated endocytosis (GO:0006898); (F) extracellular matrix organization (GO:0030198); (G) antimicrobial humoral response (GO:0019730); (H) cytokine-mediated signaling pathway (GO:0019221); (I) retinoid metabolic process (GO:0001523); (L) immune response (GO:0006955); (M) blood coagulation (GO:0007596); (N) membrane organization (GO:0061024); (O) pyruvate metabolic process (GO:0006090); (P) signal transduction (GO:0007165); (Q) chemical synaptic transmission (GO:0007268); (R) regulation of lipid metabolic process (GO:0019216); (S) transmembrane transport (GO:0055085); (T) glutamate secretion (GO:0014047).

We conducted an in-depth analysis of the protein data found in the reviewed studies, as reported in Table 1. For the 140 proteins (Table S1), we first undertook an analysis based on the matrix (e.g., blood, brain, saliva, urine, and lymphoblastoid cell lines) and on the number of times (frequency) that the protein was reported in all of the analyzed articles. The analysis showed a major presence of the apolipoproteins (APOE and APOA1 proteins) and fibronectin (FN1). Then, we analyzed all of the biological pathways associated with the proteins and clustered all of the proteins according to the biological process (BP). Interestingly, the results showed that the two major biological processes associated with ASD were platelet degranulation and lipid metabolism (Figure 4). 

Indeed, platelets play a safe role in the pathophysiology of thrombogenesis and atherogenesis, and this connection may also be due to an increase in Body Mass Index in ASD children [112]. In addition, serotonin is also present in the platelet, and stimulates aggregation by exercising the vasoconstrictor and thrombogenic effect in response to lesions of the basal endothelium. In fact, it is now known that many ASD patients have hyperserotonemia or elevated serotonin levels in whole blood (5-hydroxytryptamine, 5-HT). Despite decades of study, the mechanisms behind this well-replicated biomarker and the contributions of the serotonergic system to ASD remain unclear and further studies are necessary [113]. Almost all whole blood 5-HT is found in the platelet and the serotonin 5-HT2A receptor improves platelet functions induced by adenosine diphosphate (ADP) signaling, with exposure to phosphotidylserine (PS) and receptor activation fibrinogen. 

Overall, the 5-HT2A receptor improves platelet aggregation. Thus, this suggests some level of mutual regulation. Remarkably, this link with the serotonin 5-HT2 receptor has been extensively studied in ASD [114]. Although the importance of fats in the correct development and maintenance of cells of the nervous system is now recognized, lipids may actually play a role in regulating inflammation.

Overall, proteomics studies of ASD remain limited and vary between different technological approaches, focusing on various matrices. However, blood may be promising for diagnostic purposes, and because the data produced from the analysis are more accessible. Finally, faeces represents an intriguing matrix, mostly concerning the strong relationship between ASD and GI symptoms; however, in this case, the gut bacterial proteome/metaproteome [102] could be involved in the investigation even though, to the best of our knowledge, no metaproteomics studies have been published to date.

## 5. Metabolomics

Metabolomics is an approach to understand the metabolic pathways and metabolic network regulation of a biological system. There are therefore many fields of application of this discipline, including medicine and biology [115].

Metabolomics has been studied to identify possible biomarkers in the serum or urine samples of patients with obesity [116], diabetes [117], and coronary heart diseases [118]. Recently, it has been evidenced that perturbations of metabolic pathways could affect the pathogenesis of central nervous system disorders [119].

Thus, metabolomics provides a powerful tool to map these perturbations and their relationship to disease and response to therapy. In fact, some studies have been carried out to investigate metabolomics biomarkers in the brain [65,66], plasma [67,68,70], dried blood [79] and urine [72,73,74,75,76,77,78] samples of ASD patients (Table 2). Yap and coworkers used a proton nuclear magnetic resonance (^1^H-NMR) spectroscopy method that showed increased levels of taurine and low content of glutamate in urine samples of ASD patients [72]. Ming et al. used a combination of liquid chromatography (LC-) and gas chromatography (GC-)-based MS, and revealed abnormal amino acid metabolism and increased oxidative stress in urinary specimens of ASD patients [73].

Mavel et al. identified more than 150 metabolites in urine, comparing 30 ASD children with 28 neurotypical subjects. Increased levels of succinate, taurine, β-alanine, and glycine were found in ASD subjects [74]. In another study, a statistically significant increase of homovanillic acid, tryptophan, glycolic acid, and 3,4-dihydroxybutyric acid was detected [76]. The authors supposed that the increase in glycolate was linked with primary oxaluria type I, and that the phenomenon was also related to yeast overgrowth.

Tryptophan and homovanillic acid have been indicated as metabolites of neurotransmission probably involved in neurodevelopmental disorders. Although 3,4-dihydroxybutyric acid may be a typical component of human urine, its increase has been highlighted in cases of succinic semialdehyde dehydrogenase deficiency, which represents a disorder in patients that could also show ASD features [120]. Recently, Dieme et al. detected increased levels of indoxyl, indoxyl sulfate and *N*-acetylarginine, and decreased levels of methylguanidine and other compounds, such as desaminotyrosine and dihydrouracil [77]. Bitar et al., in a group of 40 ASD children and 40 age-matched controls, highlighted perturbations in various compounds [78], including 2-hydroxybutyrate, glutamate, creatine, and tyrosine. In addition, the authors also identified metabolites, including cysteic acid, guanine and trigonelline. These results showed abnormalities in carbohydrate and amino acid metabolisms, in addition to differences in oxidative stress pathways [78].

Furthermore, by using a GC-MS approach in urine sample analysis, it has been possible to build a multivariate statistical model that captures global biochemical signatures of autistic individuals, thus enabling patients to be distinguished from healthy children [75]. To investigate metabolites potentially related to the ASD disorder, it is possible to also study metabolites in plasma samples. The analysis of these metabolites tended to approximately support the results noted in urine, although specific gaps exist due to differences in renal clearance for some compounds.

Plasma fatty acids were recognized as diagnostic markers for ASD, specifically as an increase in the most saturated fatty acids and a decrease in polyunsaturated fatty acids, such as valeric, hexanoic and stearidonic acids [121]. Kuwabara et al. identified diverged levels of plasma metabolites connected with mitochondrial dysfunction and oxidative stress in ASD patients [67]. In a study by Wang et al., a cohort of ASD patients and participants without autism were analyzed using ultra-performance LC quadrupole time-of-flight tandem MS (UPLC/Q-TOF MS/MS) to identify metabolic variations in serum. In particular, the authors identified 17 metabolites, two of which were associated with ASD and could be significant predictors of autism: sphingosine 1-phosphate (S1P) and docosahexaenoic acid (DHA) [69].

West et al. used LC-MS/GC-MS to recognize significant concentrations in aspartate, dehydroepiandrosterone sulfate (DHEA-S), glutaric acid, serine, and succinic acid. Decreased levels of citrate, creatinine, glutamate, hydroxyphenyllactate and isoleucine were detected, thus indicating the roles of altered abnormal mitochondrial energy production (succinic acid, DHEA-S, citrate, aspartate, glutamate) and branched-chain amino acid metabolism (isoleucine, hydroxyphenyllactate) [68].

Rangel-Huerta et al. used untargeted metabolomics (HPLC–MS/MS) of plasma samples from 30 ASD children and 30 age-matched controls. In this study, ASD patients were subdivided into two groups: with (AR) and without (ANR) neurologic regression. The metabolic intermediates were detected in the aspartate, beta-alanine, glucose–alanine cycle, malate–aspartate shuttle, urea cycle, and tryptophan breakdown pathways showing significant statistical differences between controls and ASD patients. In addition, within the two subgroups, significant statistical differences were also highlighted in the levels of the fatty acids decanoylcarnitine, laurate, arachidate, myristate, octanoylcarnitine, quinate, and 7-methylurate [70]. Rachel S. Kelly et al. highlighted differences in tyrosine metabolism, tryptophan biosynthesis and endocannabinoid metabolism in a cohort of children, 13% of whom had ASDs. In addition, they hypothesized that metabolomic biomarkers could help to identify children with poor communication skills [71].

A recent study investigated the metabolic profile by extracting molecules from dried blood spots (DBSs) and performing a targeted analysis on a panel of 45 metabolites [79]. The level of nine of these molecules (20%) was significantly higher in ASD patients with respect to controls, including citrulline and acyl-carnitines C2. The results suggested that the mitochondrial fatty acid β-oxidation pathway was less active, revealing hidden molecular mechanisms related to ASD. Thus, this non-invasive methodology was shown to be suitable for a screening of newborn subjects to emphasize modifications in metabolic profiles during development [79].

Moreover, it is possible to analyze the metabolomes derived from brain tissue, for example, using LC-MS analyses for untargeted metabolomics analysis. As reported by Graham (106), in brain tissue from 11 deceased subjects with ASD, compared to 11 controls, a group of statistically significant compounds, such as 3-methoxytyramine, 5,6-dihydrouridine and *N*-carboxyethyl-γ-aminobutyric acid, was detected [65].

Post-mortem prefrontal cortex samples were analyzed by Kurochkin et al. in a cohort of 32 ASD subjects and 40 controls. The study identified increased levels of glutathione disulfide and 5-oxoproline and, on the contrary, decreased levels of glutathione, L- γ-glutamyl-cysteine and l-cysteinyl-glycine. All of these metabolites were involved in their respective metabolic pathways and in others such as pyruvate metabolism, starch and sucrose metabolism, arginine and proline metabolism and TCA cycle [66].

**Figure 5:**
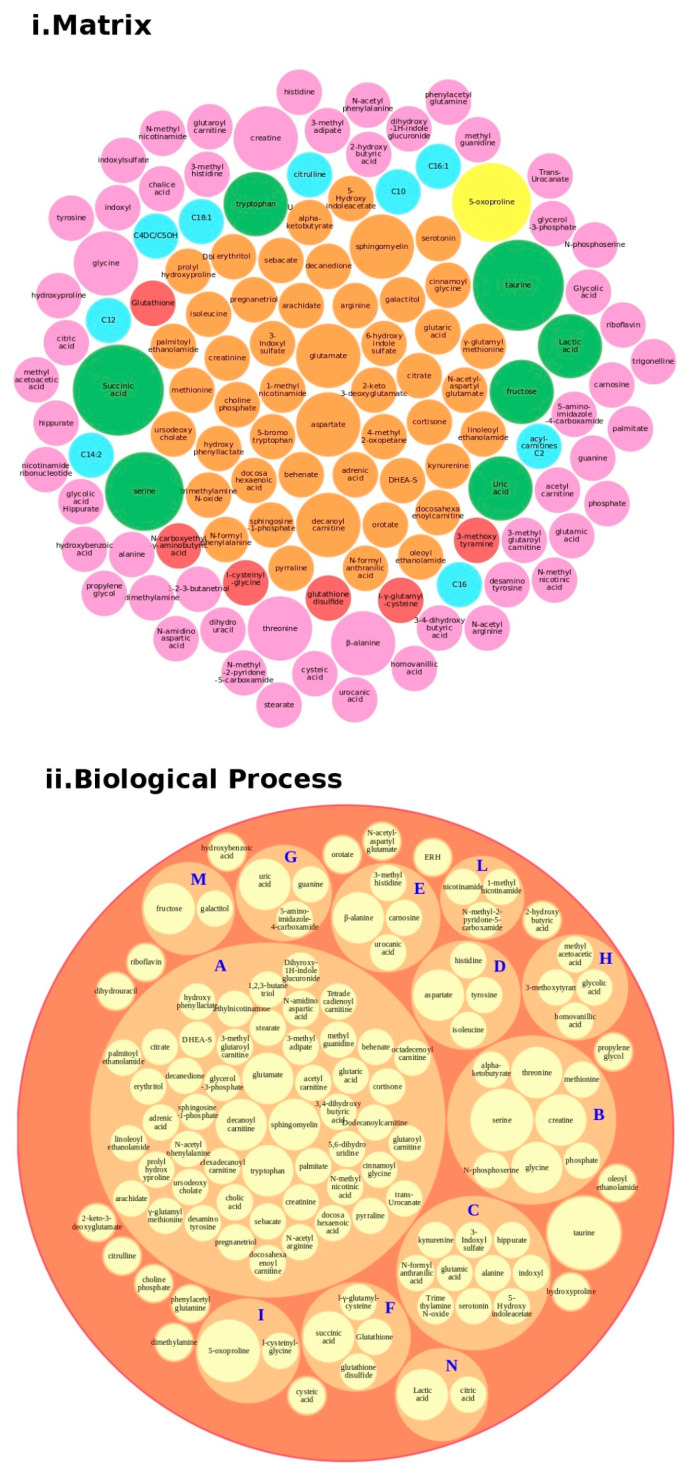
Metabolites analysis as a tool for the decision support system (DSS). In box (**i**), we grouped the metabolites highlighted in Table 2 and Table S1 according to the matrix in which the metabolites were studied, such as blood (orange), blood and urine (lawn green), brain (red), dried blood (brown), urine (pink), and brain, urine and blood (lilac). The size of the bubbles indicates the number of times the metabolites were found in that matrix in the different studies taken into consideration. We analyzed the data for the biological process (box (**ii**) and clustered the metabolites. Legend code: A: Lipid Metabolism Pathway; B: Glycine and Serine Metabolism; C: Tryptophan Metabolism; D: Transcription/Translation; E: Histidine Metabolism; F: Glutamate Metabolism; G: Thioguanine Action Pathway; H: Tyrosine Metabolism; I: Glutathione Metabolism; L: Nicotinate and Nicotinamide Metabolism; M: Galactose metabolism; N: Glutaminolysis and Cancer.

Regarding proteins, we performed a bubble analysis of the metabolites (Figure 5). For the 119 metabolites (Table 2 and Table S1), the first analyses based on the matrix showed a major presence of glutamate, decanoyl carnitine, and tryptophan, clustered in the major BP lipid metabolism pathway, confirming the data from the proteins. In addition, another implicated BP was the tryptophan metabolism, which was implicated in the gut–brain axis because of an increased 5′-HT, leading to tryptophan depletion and contributing to hyperserotonemia, which is associated with GI symptoms and neurodevelopmental disorders [47].

The study of the metabolites in the ASD subjects provides an understanding of the complexity of the system in terms of the main metabolic interactions between metabolites, with particular reference to their relationship with ASD.

## 6. Interactome in ASD

In the context of a multi-omics functional approach, the identification of proteins related to ASD, together with their functional annotation, correlations, and association to biological pathways, can be related to the metabolomics outcome, to depict a comprehensive picture of the functions involved in the ASD pathogenesis or in the identification of ASD-related biomarkers.

In this sense, interactomics, namely the ensemble of all of the molecular interactions that could take place in a group of proteins involved in a biological function, is furthermore key for data interpretation. Currently, more than 650,000 protein–protein interactions (PPIs) have been reported and found to constitute the human interactome. [122,123]. Furthermore, it was found that PPIs have been involved in most diseases, such as neurodegenerative disorders, leukemia, cervical cancer, and bacterial infection [122]. In fact, many human diseases involve the loss of an essential interaction or formation of a protein complex at an inappropriate time or location [122]. A possible role of the interactome in psychiatric diseases, such as ASD, has been hypothesized, focusing on patients who show alterations in some biological pathways, including calcium homeostasis, oxidative stress, energy metabolism, synaptic transmission, cytoskeleton, and immune system development [124,125,126].

Studies have highlighted that disruptions in the Schizophrenia 1 (DISC1) gene single-nucleotide polymorphisms are related to some psychiatric disorders, including ASD [127]. Protein complexes of DISC1 are involved in intracellular transport, cell cycle/division and cytoskeletal stability and organization [128]. Sakai et al. [129] found new interactions among protein products encoded by ASD-associated genes, including tuberous sclerosis 1 (TSC1) and tuberous sclerosis 2 (TSC2) proteins, which are implied in tuberous sclerosis complex (TSC), a rare disease associated with ASD [130].

Alfieri et al. studied the synaptic interactome associated with the p140Cap protein, which is involved in synaptogenesis, plasticity, and synaptic transmission [131]. Another study investigated the MET receptor tyrosine kinase one, as a potential interactome in ASD. MET receptor tyrosine kinase is involved in spine morphogenesis and, synaptic structures including dendritic complexity, controlling neuronal growth, functional maturation, and glutamatergic synapse maturation in the hippocampus [132].

SHANK3 gene duplications, deletions, and various point mutations have been observed in ASD patients, with the role of organizing the postsynaptic density by assembling complexes with signaling molecules, postsynaptic receptors, and cytoskeletal proteins [14,133,134,135]. ASD symptoms, such as motor hyperactivity, a tendency toward acoustic startling, reduced prepulse inhibition and abnormal circadian rhythms, were found in some SHANK3 overexpressing transgenic mice [133]. In another work, Lee et al. investigated how the rapamycin interactome could target SHANK3 in a mammalian model [136].

All of these studies provide an idea of the possible biological interactions in psychiatric disorders, such as ASD. Currently, however, information from interactomics is not sufficient to delineate a proper picture of the system. A comprehensive map could clearly help to provide crucial information for more accurate diagnosis and targeted treatments. Future investigations should consider the many factors that are often neglected, from family history and lifestyle to age, ethnicity, and dietary habits.

## 7. Clinical Decision Support Systems to Improve Medical Diagnosis on ASD

Clinical decision support systems (CDSSs) are described as “active knowledge systems that use two or more items of patient data to generate case-specific advice” [137]. Medicine is now oriented toward personalized and precise treatment. The application of electronic medical and health record systems (EHR/EMR) and integration with data from translational research will improve the timing of diagnosis and, therefore, reduce costs. Thus, CDSS is a tool that incorporates clinical patient information into big data from omics platforms to improve patient care.

The CDSS was designed to assist the clinician in the relationship with the patient from initial consultation to diagnosis and follow-up. In fact, CDSS has been shown to positively influence clinical outcomes, improving the quality of patient safety [138,139,140,141], reducing bias from medical decisions [142,143], yielding more reliable data [144,145], and promoting prevention and specific treatments [146,147,148]. For example, the Child Health Improvement through Computer Automation system (CHICA) is a computer-based CDSS developed for the automation of the management of chronic disease and their prevention. It also works as an electronic health record (EHR), supporting and easing the workflow of pediatricians [149,150].

In a clinical trial, Downs et al. used CHICA integrated with the EHR on an electronic tablet or a sheet of scanned paper that families could complete in the waiting room. CHICA analyzes the answers to the questions and selects the six most important alerts or reminders for the clinician. These results are accumulated into a visit agenda and the clinician can respond to the alerts and reminders on the agenda. Thus, this method demonstrates that automating surveillance for ASD and automating the administration of a screening test can result in high rates of screening [151].

To better understand ASD and be more able to stratify the population of ASD patients into subgroups, it is necessary to integrate all of the data from the omics with the data collected by the clinician and analyze them through machine learning models. This allows advanced models to be generated and also used in clinical practice. The wide range of generated omics data, particularly those obtained from proteomics and metabolomics analyses, requires an advanced computational analysis that allows identification of possible biomarkers associated with the different ASD phenotypes, such as high or low functioning, and presence or absence of repetitive behaviors. In turn, this enables a deeper understanding of the molecular mechanisms associated with the different phenotypes and the implementation of personalized treatments.

The increase in omics data is contributing to the use of the molecular subtype in complex diseases; in particular, this approach is already used in the study of cancer, such as for the Cancer Genome Atlas (TCGA) [152]. 

In addition to this approach, efforts are being made to implement data from omics, such as the multi-omics profiling expression database (MOPED), to process these data, and to standardize data from genomics and proteomics [153,154,155,156]. Additionally, in the field of autism, databases such as the National Database for Autism Research [157] have been created to host multidisciplinary omic data, including exome sequencing, brain imaging, and clinical diagnostic data.

These types of automation, such as the CHICA system with EHR and raw data from omics approaches, based on the computation of machine learning algorithms, are the types of approaches currently under development. Furthermore, these approaches can considerably increase the speed at which children can be screened for ASD, thus improving the age of diagnosis and helping to better define the ASD subgroup. Data from the multi-omics approach (such as that from proteomics, and metabolomics, in particular) will be elaborated with the information taken from clinician reports (such as neuropsychiatric or GI symptoms). Artificial intelligence (AI) will integrate this information to study the characteristics of ASD groups, and stratify patients into sub-groups according to the phenotype characteristics.

**Figure 6:**
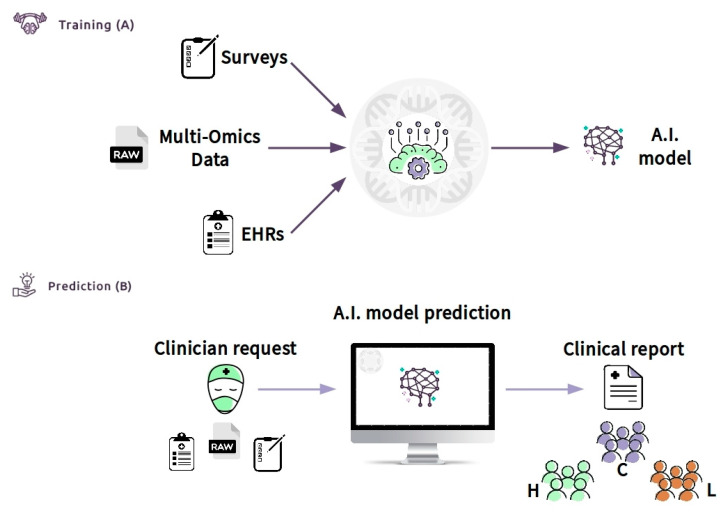
Clinical decision support system (CDSS) as a new approach to ASD children to screen and improve the age of diagnosis: Box (**A**) (training) shows the flowchart including all data used (survey, such as Child Health Improvement through Computer Automation system (CHICA), omics data and electronic medical records (EHR)) by machine learning model to classify patients. Once the model has been constructed with good accuracy, the clinician (box (**B**)) (prediction) will upload the patient data. An A.I. model will predict the class with an actionable result summarized in the clinical report. The result will generate subgroups based on the patient’s features, for example, high functioning (H), low functioning (L), and control group (C). This system might be used by clinicians to improve early diagnosis because it provides significant information about the features of the ASD patient.

Moreover, the CDS can be defined as an “expert system”, according to the classification of Wright et al. [158]. Expert systems assist clinicians by offering complex decision support by combining patient data with electronically available data. In our case, we crossed clinical data (EHR), laboratory data (multi-omics data), and lifestyle data (a survey) of patients. The CDSS can be made available through the Internet (e.g., https://cds.asd.com). The system can be trained with data of a cluster of patients of known diagnosis (Figure 6A). Then, the clinician will be able to access the web interface and upload all of the required information. The software can be connected directly to the hospital database through the laboratory information system (LIS) and can allow the collection of all of the required inputs, such as EHR information (Figure 6B). The system, consisting of bioinformatics algorithms and machine learning models, will analyze the data and provide, via the web interface, a clinical report. The results in the form of a clinical report can be easily interrogated to support other clinicians in a possible diagnosis or treatment of autism phenotypes, after deep stratification of different types. The CDSS will be able to increase accuracy with the increase of the so-called continuous learning of the machine. Thus, the expectation is that the CDSS, properly guided, will make a beneficial contribution to patient care at all levels.

## 8. Discussion and Future Prospects

It is clear that the etiology of ASD is given by a combination of genetic and environmental factors, and the only therapy for ASD that has been demonstrated to be effective is behavior analysis. Furthermore, genetic and environmental factors are implicated in changes in the brain and metabolism, such as mitochondrial dysfunction, neurotransmitters alteration, abnormal neuron development, neuroinflammation and immune dysregulation and oxidative stress. In addition, the diagnosis of ASD currently depends on clinical observation and procedures to evaluate behavioral, historical, and parent-report information [48].

There are also important ASD-related considerations to be taken into account, namely, ASD’s etiological heterogeneity, varied comorbidities, and the complexity of the brain as the centrally affected organ. In addition, there have been relatively few omics-based studies of ASD thus far. These aspects may involve significant variability, therefore, detecting proteomics and metabolomics biomarkers that look at the functional aspect may contribute to the advance of the clinical diagnosis of ASD and find new tools that could be used to estimate the outcome of individualized interventions. Results produced from proteomics-approaches show that differential expression of mitochondrial bioenergetics (involving NDUV1 protein), inflammation and immune function (MBP protein), proteins of lipid metabolism (APOB-100) and synaptic biology (SYT1 protein) are crucial in the pathogenesis and progression of the ASD. In fact, synaptic degeneration and mitochondria dysfunction are examples of the cellular events that could be correlated with ASD and cognitive impairment.

The altered metabolites are mainly associated with fatty acid metabolism (decanoyl-L-carnitine), oxidative stress (e.g., glutathione), mitochondrial dysfunction (e.g., arginine), amino acid metabolism, cholesterol metabolism, energy metabolism (e.g., Succinic acid), intestinal microbiota (e.g., tryptophan) and neurotransmitters (e.g., γ-aminobutyric acid). 

**Figure 7:**
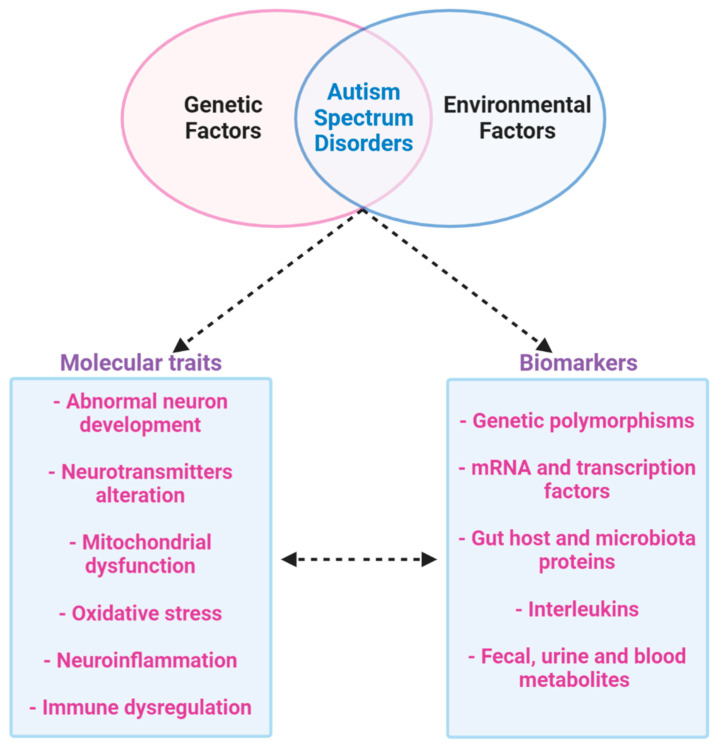
Multi-omics approach to ASD. ASD is a multifactor disease that includes genetic and environmental factors. The phenotype of ASD determines abnormal neurodevelopment with alteration in neurotransmitters. Furthermore, ASD is characterized by mitochondrial dysfunction, oxidative stress, inflammation and abnormal immune regulation. All of these dysfunctions produce possible biomarkers that could be identified by a new multi-omics approach to studying ASD.

Therefore, the results highlighted by metabolomics confirm that mitochondrial dysfunction may be a risk factor for autism; it could also be a target to find a possible biomarker to identify ASD patients and to show different expressions in subgroups of patients. All of these results may influence social and cognitive deficits in autism. In addition to elucidating the ASD pathobiology, multi-omics approaches could lead to the identification of novel biomarkers for improved diagnosis and therapeutic monitoring and diagnosis; hence the ultimate aim is to find biomarkers that are simple, inexpensive, and noninvasive, in accessible tissues and body fluids such as the blood or, preferably, urine (Figure 7).

Currently, the main limits are due to the lack of uniformity in the collection of clinical phenotypes with homogeneous methods; moreover, few studies have addressed the degree of correlation between different biomarkers or evaluated multiple biomarkers or endophenotypes in parallel. Furthermore, studies are limited in their evaluation of biomarkers by comparisons of patients with ASD and healthy controls, without considering the family and specific characteristics of the pathology. Often, the sample cohort is also highly limited. From the point of view of omics data, the biggest limit is that all of the data from the omics are not considered and the data are not integrated with collected clinical data. 

Thus, this big data approach will be essential for the early detection of ASD and the possibility of developing more precise medicine to design and optimizing the pathway for diagnosis, therapeutic intervention, and prognosis using omics data that are able to highlight individual variability between ASD patients.

In this context, proteomics and metabolomics offer the best possibility of viewing changes in the functional pathways associated with the disease, such as increases or decreases in the expression of proteins or metabolites markers. These markers can be differently expressed in different patient cohorts thus helping improve diagnostics accuracy, ASD population stratification, and the development of effective personalized treatments.

## Figures and Tables

**Table 1:** Proteomics-based targets of ASD.

Reference	Matrix	N° of Subjects	Analytical Technique	Proteins Implicated and Pathways
[50] Junaid MA et al., 2004	Brain	8 ASD and 10 controls	2-DE, LC-MS/MS	Glo1 (osteoclastogenesis and ASD etiology)
[51] Broek JA et al., 2014	Brain	prefrontal cortex, 10 ASD and 10 controls; cerebellum, 16 ASD, 17 controls	SRM-MS	VIME, CKB, MAG, MBP, MOG, PLP1, DNM2, STX1A, STXBP1, GFAP, PACSIN1, SYN2, SYT1 (synaptic transmission and energy metabolism)
[52] Wingo et al., 2019	Brain	27 ASD, 76 controls	Large-scale proteome-wide association	VGF, SEPT5, DBI, MAPT, KIAA1045, DLD, ABHD10, VDAC1, NDUFV1, PDHB (cognitive stability)
[53] Corbett et al., 2007	Blood	69 ASD and 35 controls	LC-MS/MS	FHR1, C1Q, FN1, APOB-100 (indirect impact on brain development in ASD)
[54] Ngounou Wetie et al., 2014	Blood	7 ASD and 7 controls	one dimensional gel electrophoresis (1-DE), LC-MS/MS	APOA1, APOA4, PON1 (Lipid metabolism - paraoxonase activity)
[55] Steeb et al., 2014	Blood	30 ASD and 29 controls	Immunoassay, LC-MS/MS	ADIPO, ARMC3, APOA1, APOE, APOC2, BMP6, CHGA, CLC4K, CTGF, EPO, FETUB, GLCE, ICAM1, IL3, IGA, IL16, IL12A, MRRP1, RGPD4, SHBG, PAP, PTPA, RN149, TENA, TLE1, TNF, TF, TRIPB, ZC3HE (Lipid metabolism, cell growth, inflammation)
[56] Yang et al., 2018	Blood	68 ASD and 80 controls	MALDI-TOF MS	APOC1, AFP, CPB2, FAPB1, FGA, PF4, SERPINA5, TAAR6 (lipid metabolism)
[57] Cortelazzo et al., 2016	Blood	30 ASD and 30 controls	2-DE, LC-MS/MS	A1AT, A2M, HPT, FIBB, FIBG, APOA1, APOA4, APOJ, ALBU, IGHA1, IGHAG (acute inflammatory and lipid metabolism)
[58] Feng et al., 2017	Blood	15 ASD and 15 controls	2-DE, Western blot/protein carbonylation and MALDI-TOF	C8A, IGKC (Immune system)
[59] Shen et al., 2018	Blood	30 ASD and 30 controls	Proteo-Miner protein enrichment, iTRAQ, LC-M S/MS	ACTG1, ACTN1, AGT, APOE, CALM1, CALR, C3, C5, EHD3, ENO1, FERMT3, FBLN1, FN1, IGFALS, ITGA2B, MAPRE2, PARVB, SERPINA1, SERPIN4A, THBS1, TLN1, VCL, VCP, VTN (complement system, platelet function, focal adhesions, cytoskeleton, motility and migration and synaptogenesis)
[60] Castagnola et al., 2008	Saliva	27 ASD and 23 controls	LC-MS/MS	HTN1, PRP, STATH (Antimicrobial peptides and central nervous system)
[61] Ngounou Wetie et al., 2015	Saliva	6 ASD and 6 controls	2-DE, LC-MS/MS	AMY1A, AZGP1, CREBBP, CST5, FRAT1, GRTP1, KIF14, ITGA6, HERC1, MRP14, MUC16, PLG, PSP, PIP, TF, ZG16 (immune and inflammation response, lipid metabolism, oxidative stress)
[62] Ngounou Wetie et al., 2015	Saliva	6 ASD and 6 controls	LC-MS/MS	DMBT1, ELANE, HTN1, IGKC, IGHG1, IGLC2, LTF, PIGR, PIP, PRH1, STATH (Inflammation and Immune response)
[63] Suganya et al., 2015	Urine	30 ASD and 30 controls	2-DE, MALDI-TOF MS	IGHG1, KNG1, MASP2 (Inflammation, coagulation and complement system)
[64] Pichitpunpong et al., 2019	lymphoblastoid cell lines	Not available	2-DE, LC-MS/MS, Western blotting	DLD, IDH2, TPT1, ANXA5, CCT5, COX5A, LGALS1, GSTP1, HNRNPA1, PGAM1, TUBB, H3F3C, DBI, AHSG, ERH, CLTA, CALM1, ENO1 (Neurological functions and inflammation)

**Table 2:** Metabolomics-based targets of ASD.

Reference	Matrix	N of Subjects	Analytical Technique	Metabolite Implicated and Metabolic Process
[65] Graham et al., 2016	Brain	11 ASD and 11 controls	LC-LTQ Orbitrap MS	3-methoxytyramine, 5,6-dihydrouridine, N-carboxyethyl-γ-aminobutyric acid (dopamine, nucleotide metabolism and cell growth and proliferation)
[66] Kurochkin et al., 2019	Brain	32 ASD and 40 controls	UPLC–MS/MS	5-oxoproline, glutathione disulfide, Glutathione, l-γ-glutamyl-cysteine, l-cysteinyl-glycine (glutathione; purine; pyruvate; propanoate; TCA cycle; galactose; starch and sucrose; nicotinate and nicotinamide; cysteine and methionine; arginine and proline metabolism)
[67] Kuwabara et al., 2013	Blood	25 ASD and 28 controls	CE-TOF MS	arginine, taurine, 5-Oxoproline, lactic acid (oxidative stress and mitochondrial dysfunction)
[68] West et al., 2014	Blood	52 ASD and 30 controls	LC-MS and GC-MS	aspartate, DHEA-S, glutaric acid, serine, succinic acid citrate, creatinine, isoleucine, hydroxyphenyllactate, glutamate (energy production, mitochondrial disease or dysfunction and oxidative stress)
[69] Wang et al., 2016	Blood	173 ASD and 163 controls	UPLC/Q–TOF– MS/MS	decanoylcarnitine, pregnanetriol, adrenic acid, docosahexaenoic acid, sphingosine-1-phosphate, uric acid (β-oxidation, fatty acid and mitochondrial dysfunction)
[70] Rangel-Huerta et al., 2019	Blood	20 ASD and 30 controls	UPLC–MS/MS	1-methylnicotinamide, 3-Indoxyl sulfate, 4-methyl-2-oxopetane, 5-bromotryptophan, 6-hydroxyindole sulfate, cortisone, methionine, tryptophan, γ-glutamylmethionine, ursodeoxycholate, sphingomyelins, kynurenine, choline phosphate, decanoylcarnitine, 2-keto-3-deoxyglutamate, arachidate, behenate, fructose, sebacate, dodecanedioate, glutamate, aspartate, orotate, galactitol, N-acetyl-aspartyl glutamate (amino acid, lipid, nicotinamide)
[71] Kelly RS et al., 2019	Blood	403 children of which 52 ASD	UPLC-MS / MS	trimethylamine N-oxide, cinnamoylglycine, linoleoyl ethanolamide, palmitoyl ethanolamide, erythritol, docosahexaenoylcarnitine, prolylhydroxyproline, alpha-ketobutyrate, serotonin, oleoyl ethanolamide, N-formylphenylalanine, 5-Hydroxyindoleacetate, pyrraline, sphingomyelin, N-formylanthranilic acid (Endocannabinoid metabolism, Xenobiotics, Tryptophan metabolism, Urea cycle, Amino acid mebolism, Fatty acid metabolism, Phospholipid metabolism, Sphingolipid metabolism)
[72] Yap et al., 2010	Urine	39 ASD and 34 controls	^1^H-NMR	N-methylnicotinamide, N-methylnicotinic acid, dimethylamine, succinic acid, taurine, N-methyl-2-pyridone-5-carboxamide, hippurate, phenylacetylglutamine, platelet serotonin (gut microbiota and gastrointestinal dysfunction)
[73] Ming et al., 2012	Urine	48 ASD and 53 controls	UPLC–MS/MS and GC-MS	trans-Urocanate, glutaroylcarnitine, 3-methylglutaroylcarnitine, β-alanine, alanine, carnosine, glycine, histidine, serine, threonine, taurine, uric acid (oxidative stress; amino acid; mammalian microbial co-metabolism)
[74] Mavel et al., 2013	Urine	30 ASD and 28 controls	2D-NMR	serotonin, glycine, β-alanine, taurine, succinic acid, creatine, 3-methylhistidine (dopaminergic, serotonergic, synapse, tryptophan, oxidation, amino acids metabolism)
[75] Emond et al., 2013	Urine	26 ASD and 24 controls	GC-MS	succinic acid, glycolic acid, hippurate, phosphate, palmitate, stearate, 3-methyladipate (gut microbiota)
[76] Noto et al., 2014	Urine	21 ASD and 21 controls	GC-MS	3,4-dihydroxybutyric acid, glycolic acid, homovanillic acid, tryptophan 1,2,3-butanetriol, fructose, propylene glycol (interactions among diet, gut microbiota and host genetics)
[77] Diémé et al., 2015	Urine	30 ASD and 32 controls	^1^H-NMR, 2D-HSQC NMR, LC-HRMS	N-acetylarginine, indoxyl, indoxylsulfate, dihydroxy-1H-indole glucuronide, methylguanidine, desaminotyrosine, dihydrouracil (gut microbiota)
[78] Bitar et al., 2018	Urine	40 ASD and 40 controls	^1^H-NMR, 2D-HSQC NMR and LC-HRMS	5-amino-imidazole-4-carboxamide, chalice acid, glutamic acid, N-phosphoserine, nicotinamide ribonucleotide, glycerol-3-phosphate, trigonelline, riboflavin, 2-hydroxybutyric acid, 5-oxoproline, acetylcarnitine, cysteic acid, citric acid, threonine, creatine, serine, N-acetylphenylalanine;tyrosine, hydroxybenzoic acid, hydroxyproline, lactic acid, guanine, N-amidino aspartic acid, methyl acetoacetic acid, urocanic acid (amino acids; carbohydrates and oxidative stress)
[79] Barone et al., 2018	Dried Blood	83 ASD and 79 controls	ESI-Tandem MS/MS system	citrulline, acetylcarnitine, methylmalonyl/3-OH isovalerylcarnitine, decanoylcarnitine, dodecanoylcarnitine, tetradecadienoylcarnitine, hexadecanoylcarnitine, octadecenoylcarnitine (amino acids, fatty acid, gastrointestinal disturbances and mitochondrial dysfunction)

## Supplementary Materials

The following are available online at https://www.mdpi.com/1422-0067/21/17/6274/s1, Table S1: Table of all proteins with UniProt code with associated matrix.

## Abbreviations

1-DE	one-dimensional gel electrophoresis
2-DE	two-dimensional gel electrophoresis
5′-HT	5-hydroxytryptamine
ADI-R	Autism Diagnostic Interview-Revised
ANR	without neurologic regression
APO	apolipoprotein
AR	neurologic regression
ASD	Autism Spectrum Disorders
AUC	area under the ROC curve
BBB	Blood–brain barrier
CDSS	Clinical decision support system
CHD8	chromodomain helicase DNA binding protein 8
CHICA	Child Health Improvement Trough Computer Automation
CNTNAP2	Contactin associated protein-like 2
DB	protein databases
DBI	diazepam-binding inhibitor
DBS	dried blood spots
DHA	docosahexaenoic acid
DHEA-S	Dehydroepiandrosterone sulfate
DIA	Data-independent analysis
DISC1	Disrupted in Schizophrenia 1
DSS	Decision support systems
EC	external calibration
EHR	electronic health record
ELISA	enzyme-linked immunoadsorbent assay
FHR1	complement factor H-related protein
FN1	fibronectin 1
GC	gas chromatography
GI	Gastrointestinal symptoms
Glo1	glyoxalase 1
GSH	glutathione
^1^H-NMR	proton nuclear magnetic resonance spectroscopy
HPLC-MS/MS	high-performance liquid chromatography with tandem mass spectrometry
IC	internal calibration
ID	identification
IPA	Ingenuity Pathway Analysis
IS	internal standards
LC	liquid chromatography
LC-MS/MS	liquid chromatography-tandem mass spectrometry
MS	mass spectrometry
NGS	Next-generation sequencing
NMR	nuclear magnetic resonance spectroscopy
PPIs	Protein–protein interactions
ROC	receiver operating characteristic
S1P	sphingosine 1-phosphate
SH3	SRC Homology 3 Domain
SHANK 3	SH3 and multiple ankyrin repeat domain 3
SIL	Synthetic stable isotope labelled
SRM-MS	selected reaction monitoring mass spectrometry
SRM/MRM	selected reaction monitoring/multiple reaction monitoring
SRS	Social Responsiveness Scale
SWATH	Sequential Window Acquisition of all Theoretical Mass Spectra
TJ	tight junction
TSC	tuberous sclerosis complex
TSC1	tuberous sclerosis 1
TSC2	tuberous sclerosis 2
tSIL	synthetic labeled tryptic peptides
UPLC/Q-TOF MS/MS	ultra-performance liquid chromatography quadrupole time-of-flight tandem mass tandem mass spectrometry
